# Building in vitro models of the brain to understand the role of *APOE* in Alzheimer’s disease

**DOI:** 10.26508/lsa.202201542

**Published:** 2022-09-27

**Authors:** Rebecca L Pinals, Li-Huei Tsai

**Affiliations:** 1 Picower Institute for Learning and Memory, Massachusetts Institute of Technology, Cambridge, MA, USA; 2 Department of Brain and Cognitive Sciences, Massachusetts Institute of Technology, Cambridge, MA, USA; 3 Broad Institute of Harvard and MIT, Cambridge, MA, USA

## Abstract

Alzheimer’s disease (AD) is a devastating, complex, and incurable disease that represents an increasingly problematic global health issue. The etiology of sporadic AD that accounts for a vast majority of cases remains poorly understood, with no effective therapeutic interventions. Genetic studies have identified AD risk genes including the most prominent, *APOE*, of which the ɛ4 allele increases risk in a dose-dependent manner. A breakthrough discovery enabled the creation of human induced pluripotent stem cells (hiPSCs) that can be differentiated into various brain cell types, facilitating AD research in genetically human models. Herein, we provide a brief background on AD in the context of *APOE* susceptibility and feature work employing hiPSC-derived brain cell and tissue models to interrogate the contribution of *APOE* in driving AD pathology. Such models have delivered crucial insights into cellular mechanisms and cell type–specific roles underlying the perturbed biological functions that trigger pathogenic cascades and propagate neurodegeneration. Collectively, hiPSC-based models are envisioned to be an impactful platform for uncovering fundamental AD understanding, with high translational value toward AD drug discovery and testing.

## Abstract (precis)

Human induced pluripotent stem cell (hiPSC)–based models of the brain will be key to unraveling the role of *APOE* ɛ4 in the interconnected cellular changes underlying Alzheimer’s disease.

## Introduction

Alzheimer’s disease (AD) persists as a debilitating and widespread neurodegenerative disorder, with over 55 million people worldwide currently living with AD or a related form of dementia (Alzheimer’s Disease International, 2020; World Health Organization, 2022). AD is characterized by progressive cognitive and functional decline, in parallel with brain cell dysfunction and death (Alzheimer’s & Dementia, nd; Montine et al, 2012; Knopman et al, 2021). Early-onset familial AD begins to manifest in individuals within the range of ∼30–60 yr of age, in comparison to late-onset sporadic AD that typically develops later in life at ≥65 yr of age (Lambert et al, 2013; National Institute on Aging, 2019). Disease-causing mutations leading to familial AD have become well-established, although this form of AD constitutes only 1–5% of all cases (Reitz & Mayeux, 2014). Work in the 1990s identified the central role of amyloid-β (Aß) in familial AD arising from mutations or duplications in the genes *APP*, *PSEN1*, and *PSEN2* (Goate et al, 1991; Levy-Lahad et al, 1995; Rogaev et al, 1995; Sherrington et al, 1995; Tanzi & Bertram, 2005). In general, the Aß peptide is released from neurons via sequential proteolytic processing of the membrane-immobilized amyloid precursor protein (APP) by secretase enzymes (Haass et al, 2012). In the amyloidogenic pathway, β-secretase first cleaves APP at the ectodomain, followed by γ-secretase at the intramembrane site, liberating Aß peptides including Aß-40 and Aß-42 (among other peptide lengths). This contrasts with the physiologically normal pathway in which α- then γ-secretases consecutively cleave APP, shedding the shorter Aß-40 species. The genetic modifications underlying familial AD alter the structures of APP (encoded by *APP*; including near the secretase cleavage sites) and the γ-secretase complex (the catalytic subunit of which is encoded by *PSEN1* and *PSEN2*). As a result, there is elevated generation of the Aß-42 species, which is more prone to aggregate into neurotoxic plaques. This sequence of findings became formative work toward the neuron-centric amyloid hypothesis of AD, whereby accumulation of Aß peptide aggregates in the brain is postulated to drive other AD pathologies, including neurofibrillary tangles of hyperphosphorylated tau (p-tau) protein inside of neurons and, ultimately, neurodegeneration (Hardy & Higgins, 1992; Hardy & Selkoe, 2002). Although a relative ratio of longer to shorter Aß peptides (often the Aß-42/40 ratio) has become a more widely accepted AD biomarker (Tanzi & Bertram, 2005; Selkoe & Hardy, 2016; Hampel et al, 2021), it is worth noting that not all familial AD-causing mutations lead to increased relative or absolute Aß-42 production, including many *PSEN1* mutations that impair net γ-secretase activity and thus reduce production of both Aß species (Sun et al, 2017). More broadly, the linear causal structure of the amyloid hypothesis, with the consequent use of Aß species as biomarkers, has suffered from heightened criticism because of failing AD drugs and contradictory findings (Herrup, 2015; Makin, 2018; Panza et al, 2019; Rabinovici, 2021).

Sporadic AD accounts for over 95% of all cases, yet the exact mechanism by which this form of AD arises is still unknown (Reitz & Mayeux, 2014). Based on the understanding of familial AD, research has historically explored the formation of amyloid plaques and tau tangles as key pathological features shared by both AD forms (Tanzi & Bertram, 2005; Serrano-Pozo et al, 2011; Knopman et al, 2021). The cascade of neurodegenerative effects associated with amyloid aggregation suggests that reducing Aß load in the brain could slow or halt cognitive decline. Despite intense efforts in drug development targeting these pathological hallmarks by means of anti-amyloid antibodies and secretase inhibitors, there is no cure for AD; current therapeutic strategies provide only modest relief or yield favorable biomarker changes in the absence of a clinical response (Huang & Mucke, 2012; Canter et al, 2016; Karran & De Strooper, 2022).

In contrast to the recognized genetic changes underlying familial AD, sporadic AD is seemingly driven by a multifactorial combination of genetic and environmental influences. Age remains the most significant risk factor for developing AD (Knopman et al, 2021). Sporadic AD carries an estimated heritability over 50% (Sims et al, 2020), with genome-wide association studies (GWAS) continuing to reveal key genetic loci that modify risk (Lambert et al, 2013; Jansen et al, 2019; Kunkle et al, 2019; Wightman et al, 2021). In particular, the importance of *APOE* ɛ4 was first identified several decades ago and is now accepted to represent the single largest genetic determinant of AD (Corder et al, 1993; Saunders et al, 1993; Strittmatter et al, 1993; Knopman et al, 2021). Even with *APOE* displaying only partial penetrance, the imparted risk is significant because the ɛ4 allele is observed at relatively high frequency in the human population. GWAS analyses have impelled a shift to recognize the involvement of multiple genetic factors across different brain cell types in driving AD. However, the interplay of these genetic nodes and corresponding cell type–specific roles require further study (De Strooper & Karran, 2016).

Disentangling the complex causes of AD relies on the development and use of experimental models that recapitulate essential facets of the human brain in the healthy versus diseased state. Animal models have served as the standard platform for the study of AD and other human diseases, offering an integrated system (i.e., connected nervous to other systems, with an immune component) that can undergo controlled manipulation (Elder et al, 2010; Götz et al, 2018). Knowledge of disease-causing mutations facilitates development of animal models, as has been the case for the less common but more genetically tractable familial form of AD. For example, transgenic mouse models have provided a route to study familial AD by overexpression of human genes carrying disease-causative mutations that promote amyloid aggregation (Hsiao et al, 1996; Sasaguri et al, 2017; Götz et al, 2018). More recently, targeted gene-editing to add humanized, pathogenic mutations to endogenous risk-factor loci (e.g., *APP* and *APOE*) has rendered more physiologically relevant mouse models (Saito et al, 2014; Sasaguri et al, 2017; Götz et al, 2018; Scearce-Levie et al, 2020). Yet, fundamental biological differences exist between animal and human systems that hinder modeling of complex, human-specific neurodegenerative diseases (Drummond & Wisniewski, 2017; Sasaguri et al, 2017; Wan et al, 2020). Postmortem brain tissues from human donors capture the relevant biology, though only present a static endpoint. Consequently, such tissues do not provide a dynamic model for tracking changes before onset and during the disease nor for experimental interventions that could alter the course of disease (Serrano-Pozo et al, 2011; Lovett et al, 2020). Human cells can be extracted and grown in culture; however, such cells are difficult to isolate from the brain and lack the relevant microenvironment of a three-dimensional tissue (Abud et al, 2017; Lovett et al, 2020). Recent efforts have leveraged advances in stem-cell biology to build in vitro models of the human brain. In 2007, Yamanaka and his team described groundbreaking work in which human induced pluripotent stem cells (hiPSCs) can be derived from more readily accessible patient skin cells and reprogrammed to an embryonic-like, pluripotent state (Takahashi et al, 2007). This paradigm was soon extended to somatic cells from other donor tissues, including peripheral blood cells (Loh et al, 2010; Seki et al, 2010; Staerk et al, 2010). The hiPSCs can then be differentiated into various cell types, such as those of the brain. Accordingly, hiPSC technology has enabled the modeling of various aspects of human brain tissue in the context of Alzheimer’s disease (Penney et al, 2020; Blanchard et al, 2022; Bubnys & Tsai, 2022). Such developments are promising and crucial toward deconvoluting cell-specific roles and tissue-level features as a function of genetic and environmental factors driving AD.

**Figure 1.**
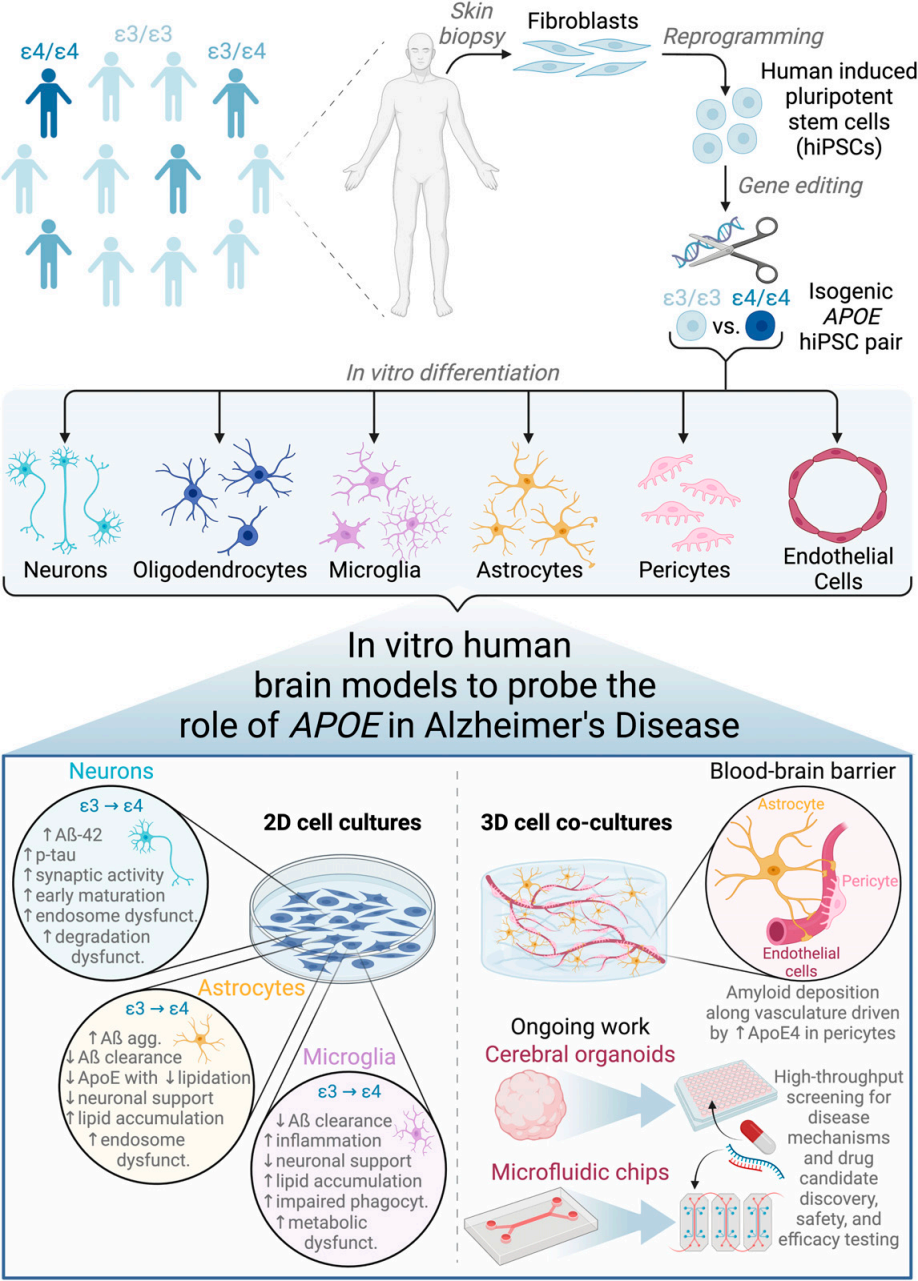
Employing human induced pluripotent stem cell (hiPSC)–based cellular and tissue models to deconvolute the function of *APOE* in Alzheimer’s disease. hiPSCs are derived from human patients of varying genetic background, gene-edited to create isogenic pairs, and differentiated into various cell types of the brain. hiPSC-based cell cultures can be formulated in conventional 2D monoculture or novel 3D co-culture geometries, the latter of which better recapitulates facets of human brain structure and function. Cell type–specific findings as detailed in the main text are summarized from 2D culture studies. Ongoing work will expand the use of cerebral organoids to modeling more diverse cell types beyond neurons and astrocytes and implement perfusable vasculature in microfluidic chip-based models of the blood–brain barrier. Such 3D co-culture models will be advantageous to both fundamental mechanistic studies to understand AD and translation into high-throughput therapeutic discovery and testing pipelines. Figure was created with BioRender.com.

In this review, we provide an overview of hiPSC-derived brain cellular and tissue models, highlighting recent work that employs these models to understand the role of the *APOE* ε4 genetic risk factor in AD (Fig 1). We begin with a brief background on how the *APOE* ε4 genotype is implicated in AD. Next, we describe foundational work in hiPSC-based brain cell modeling and then focus on findings from hiPSC-based AD models. We feature new work integrating multiple cell types and/or three-dimensional brain tissue culture systems to model AD, including cerebral organoids and engineered tissues, and conclude with outstanding challenges the field faces.

### *APOE* in Alzheimer’s disease

The significance of the *APOE* gene in governing AD risk was initially recognized in the 1990s, with a series of pioneering studies providing the crucial genotype-to-pathology association and evidence of the physical protein-to-biomarker interaction (Corder et al, 1993; Saunders et al, 1993; Strittmatter et al, 1993). *APOE* encodes the protein apolipoprotein E (ApoE). Three common forms of the *APOE* gene exist across the human population: *APOE* ε2, ε3, and ε4 (Holtzman et al, 2012). The *APOE* ɛ4 genotype has become well established as the primary genetic risk factor for developing AD through a series of independent studies and datasets across the globe (Farrer et al, 1997; Lambert et al, 2013; Yamazaki et al, 2019). Although the ε4 allele increases risk of developing AD, the ε2 allele is protective (Corder et al, 1993; Saunders et al, 1993; Holtzman et al, 2012; Reiman et al, 2020). *APOE* confers susceptibility in a dose-dependent manner: relative to an individual with the most common *APOE* ε3/ε3 background, individuals heterozygous for *APOE* ε4 (ε4/ε3) are subjected to a 2–4 times greater risk of developing AD and individuals homozygous for *APOE* ε4 (ε4/ε4) rise to an 8–15 times greater chance of developing AD (Corder et al, 1993; Farrer et al, 1997; Genin et al, 2011; Holtzman et al, 2012). In contrast, a single *APOE* ε2 allele (together with the common ε3) decreases the odds ratio to ∼0.6 (Corder et al, 1994; Farrer et al, 1997; Holtzman et al, 2012; Reiman et al, 2020). Moreover, the age of AD onset scales inversely with *APOE* ε4 allele dose, whereby each additional ε4 allele shifts the individual toward a younger age of disease manifestation (estimated 2–5 or 5–10 yr earlier for one or two copies of the ε4 allele, respectively, relative to the lower risk group) (Corder et al, 1993; van der Lee et al, 2018; Yamazaki et al, 2019). For context of the gene prevalence, anywhere from 9 to 25% of humans carry at least one copy of *APOE* ε4, with this allele frequency varying widely among population groups (Farrer et al, 1997; Bertram et al, 2007; National Institute on Aging, 2019; Yamazaki et al, 2019). However, one or two copies of ε4 is neither necessary nor sufficient to cause AD.

Following the recognition of *APOE* as a principal genetic determinant in AD, the encoded protein ApoE has become the subject of intense investigation. Yet, the exact functional connection between the polymorphic protein and ensuing AD pathologies remains elusive. ApoE is a 34-kD protein that is broadly involved in lipid metabolism, existing distinctly in the periphery and in the brain (with highest expression in the liver, followed by the brain) (Mahley, 1988; Kim et al, 2009; Holtzman et al, 2012). Within the brain, ApoE represents the most abundantly produced apolipoprotein type and is primarily made by astrocytes under physiological conditions, with lesser contributions from mural cells of the vasculature, damage-associated or neurodegenerative disease-associated microglia, and stressed neurons (Boyles et al, 1985; Pitas et al, 1987; Xu et al, 2006; Casey et al, 2015; Gosselin et al, 2017; Keren-Shaul et al, 2017; Wadhwani et al, 2019; Mahan et al, 2022). ApoE is a component of the high-density lipoprotein–like particles unique to the brain, which adopt a more discoidal morphology compared with those outside the brain (Pitas et al, 1987; LaDu et al, 1998; Holtzman et al, 2012). Similar to its role in the periphery, ApoE in the brain serves as a ligand in receptor-mediated endocytosis of these lipoprotein particles, facilitating transport of phospholipids and cholesterol to neurons (Kim et al, 2009; Holtzman et al, 2012; Yamazaki et al, 2019). Interestingly, ApoE has been found within plaques in human and transgenic mouse brains (Namba et al, 1991; Wisniewski & Frangione, 1992) and has been demonstrated to bind to the Aß peptide (Strittmatter et al, 1993), albeit in acellular experiments with synthetic proteins at above-physiological concentrations.

The three common *APOE* genetic variants result in distinct amino acid substitutions within the coding sequence of the protein: ApoE ε2 (ApoE2) has cysteines at amino acid positions 112 and 158, ApoE ε3 (ApoE3) has cysteine at position 112 and arginine at position 158, and ApoE ε4 (ApoE4) has arginines at positions 112 and 158. These single–amino acid substitutions strikingly alter the ApoE protein structure, specifically in the receptor-binding domain of ApoE2 and the lipid-binding domain of ApoE4. These conformational differences modify the corresponding protein function: the ApoE2 isoform is severely deficient in its ability to bind to the LDL receptor (<2% receptor binding activity in comparison to ApoE3), and the ApoE4 isoform exhibits a lower lipidation state and lower binding affinity to Aß (Ruiz et al, 2005; Kim et al, 2009; Mahley et al, 2009; Holtzman et al, 2012). Expression of *APOE* ε4 is reported to cause increased Aß aggregation within and impaired clearance out of the brain, in addition to other processes including synaptic dysfunction and neuroinflammation (Kim et al, 2009; Castellano et al, 2011; Holtzman et al, 2012; Knopman et al, 2021). ApoE thus maintains a clear biological association to AD, yet the multifaceted mechanism by which ApoE4 contributes to AD pathogenesis requires further study.

As *APOE* ε4 has become firmly entrenched as the strongest genetic factor predisposing individuals to sporadic AD, GWAS studies have expanded in attempt to identify other genes with such significant effects on AD risk. Despite a growing list of such genetic variants, the field increasingly recognizes that these other genes most likely operate interactively with both each other and nongenetic factors, further complicating the story (Kunkle et al, 2019; Mathys et al, 2019; Knopman et al, 2021). Harboring individual risk genes may only confer a minor heritable AD risk but become problematic when existing in certain combinations of multiple, common polymorphisms and/or with a single, rarer genetic variant.

The ability to test the functional consequences of predicted risk factor combinations has been a critical step toward understanding individual genetic contributions. This effort has been enabled by the development and use of appropriate AD models, together with the emergence of larger genomic datasets and more advanced characterization methods necessary to assess functional outputs of the systems under study. A host of animal models exist to recapitulate various aspects of AD, including a growing list of transgenic and genetically modified mice (Drummond & Wisniewski, 2017; Götz et al, 2018). Although these animal models can exhibit certain phenotypes similar to those of human AD patients, the underlying mechanisms are often quite disparate (Götz et al, 2018; Scearce-Levie et al, 2020; D’Avanzo et al, 2015). For example, a popular mouse model (5xFAD) produces high levels of Aß-42 by overexpression of human *APP* (with three AD-associated mutations) and *PSEN1* (with two AD-associated mutations). Although this model develops some pathological AD phenotypes, it has suffered from poor clinical translation. Notably, a third of putative AD risk genes identified in humans lack adequate mouse orthologs, and of particular importance, the *APOE* polymorphism does not exist in rodents (Mancuso et al, 2019). As will be described, recent work with hiPSC-based models has underscored that human and rodent glia differ significantly in terms of morphology, function, and gene expression profiles (Preman et al, 2021), particularly in the lack of *APOE* ε4-driven lipid metabolic dysregulation pathways now generally accepted to contribute to AD (TCW et al, 2022). As such, there is a growing movement in the field to take advantage of hiPSC-based models to examine the fundamental disease mechanisms occurring at the molecular and cellular scales within a genetically human background.

### Pluripotent stem cell-based models of neurological disease

The scientific breakthrough of generating human iPSCs from somatic cells was first described in 2007, wherein adult human dermal fibroblasts obtained from simple skin biopsies were reprogrammed into stem cells (Takahashi et al, 2007). From this initial discovery, there are now methodologies to differentiate hiPSCs into individual cell types of widely varying identities and organ-like cellular aggregates known as organoids. Herein, we will focus on brain-centric hiPSC-based models. Published protocols exist to derive all major cell types of the brain from hiPSCs: neurons (of different subtypes) (Yeo et al, 2007; Chambers et al, 2012; Zhang et al, 2013), oligodendrocytes (Hu et al, 2009; Wang et al, 2013; Douvaras et al, 2014), microglia (Muffat et al, 2016; Abud et al, 2017; Guttikonda et al, 2021), astrocytes (Shaltouki et al, 2013; TCW et al, 2017), pericytes (Patsch et al, 2015; Kumar et al, 2017), and endothelial cells (Lippmann et al, 2012; Patsch et al, 2015; Qian et al, 2017; Lu et al, 2021b). These differentiation protocols continue to be refined, resulting in brain cells that more accurately represent the requisite genetic expression profiles, functions, and morphologies and at higher yield and purity (Anderson et al, 2021; Lu et al, 2021a). In vivo chimeric models established by transplantation of hiPSC-derived cells into mouse brains has provided another route for producing particular cell populations and for studying neurodegenerative disease, wherein organismal integration provides cell type heterogeneity and an extracellular environment that can drive biologically relevant cell identity and AD phenotypes (Espuny-Camacho et al, 2017; Hasselmann et al, 2019; Najm et al, 2020). Rather than differentiating individual cell types, organoids are three-dimensional models that leverage early developmental programs to drive hiPSCs into self-organized tissue, often with numerous cell types present (Clevers, 2016; Kim et al, 2020; Hofer & Lutolf, 2021). Foundational work by Lancaster et al (2013) described the creation of an in vitro model of the human brain, termed cerebral organoid, and its application to model neurodevelopment and neurological disorders (Lancaster et al, 2013). Namely, the authors generated cerebral organoids to model microcephaly and determined that premature neuronal differentiation underlies the disease phenotype. This work was crucial as a proof-of-principle demonstration for modeling human diseases using patient-derived hiPSCs, showing that key features of the highly complex human brain, such as regional organization, can be emulated in a simplified organoid context.

Numerous groups have translated these hiPSC-based models to study AD over the past decade. A study that conducted neuronal differentiation of hiPSCs from patients with familial AD, sporadic AD, and control individuals highlighted the utility of this stem cell technology in recapitulating some AD-relevant phenotypes, including elevated levels of active kinase GSK-3ß that can phosphorylate tau and the accumulation of early endosomes in neurons (Israel et al, 2012). These findings have been elaborated upon with an orthogonal approach of using three-dimensionally differentiated neuronal cells originating from immortalized human neural stem cells containing familial AD mutations (Choi et al, 2014). Organoids and other three-dimensional neural tissues grown from familial AD patient–derived hiPSCs have been shown to spontaneously develop key pathological features of AD (Bubnys & Tsai, 2022), including accumulation of amyloid plaque– and tau tangle–like structures (Raja et al, 2016; Gonzalez et al, 2018; Jorfi et al, 2018), endosome abnormalities (Raja et al, 2016), and hyperexcitability (Ghatak et al, 2019). Importantly, these AD phenotypes arose in hiPSC-derived cultures in a matter of weeks to months, rather than decades for the disease to manifest in patients. In addition, these model systems supported drug response studies with secretase inhibitors, which limit the production of toxic Aß species (Israel et al, 2012; Choi et al, 2014; Raja et al, 2016; Jorfi et al, 2018). More detailed findings from hiPSC-based familial AD models have recently been reviewed elsewhere (Lee et al, 2020; Penney et al, 2020; Raman et al, 2020). Such advances are promising toward extending this framework to model sporadic AD with more diverse brain cell types present.

Comparison of hiPSC-derived cells sourced from healthy versus diseased individuals continues to be an important route for building hiPSC-based models of the brain. More recently, the CRISPR/Cas9 gene-editing system (among others) has been employed, allowing introduction of mutations into healthy hiPSCs or, conversely, correction of mutations (Ran et al, 2013; Doudna & Charpentier, 2014; Paquet et al, 2016). Accordingly, individual genetic contributions to AD risk can be deconvoluted within otherwise genetically identical (i.e., isogenic) sets of hiPSC-based cellular and tissue models. In the context of AD, this gene-editing approach has been implemented by generating panels of isogenic hiPSCs harboring familial (Konttinen et al, 2019; Kwart et al, 2019; Schrauben et al, 2020) and sporadic AD mutations, with examples of the latter detailed in the following section.

Although hiPSC-based cultures are powerful in vitro models that capture features of brain development and dysfunction, we also must acknowledge their limitations before discussing conclusions ascertained from them. For two-dimensional cell culture systems, the simplified monolayer geometry often results in monomorphic cell populations unable to capture the cell-level heterogeneity and tissue-level architectural complexity inherent in the brain (D’Avanzo et al, 2015; Grenier et al, 2020; Lovett et al, 2020; Blanchard et al, 2022). Likewise, such systems inherently lack a three-dimensional microenvironment that supports the cellular interactions and spatial context necessary to model extracellular dynamics, such as protein aggregation events (D’Avanzo et al, 2015; Grenier et al, 2020; Lovett et al, 2020). Cerebral organoids present a more relevant interstitial environment, but they often lack control and consistency in composition and spatial structuring (Di Lullo & Kriegstein, 2017; Gonzalez et al, 2018; Grenier et al, 2020; Hofer & Lutolf, 2021). Moreover, organoids frequently suffer from necrotic cores because of the lack of vascularization to locally deliver the oxygen and nutrients necessary to sustain growth (Giandomenico & Lancaster, 2017; Mansour et al, 2018; Grenier et al, 2020). The absence of blood vessels is problematic in light of the key role that vascular pathology plays in the two most common neurodegenerative diseases: AD and vascular dementia (Blanchard et al, 2020; Grenier et al, 2020). More generally, there are intrinsic drawbacks in current hiPSC-based models achieving sufficient tissue maturity and cellular diversity (Camp et al, 2015; Di Lullo & Kriegstein, 2017; Bhaduri et al, 2020; Grenier et al, 2020), in addition to losing epigenetic modifications through the reprogramming process (Maherali et al, 2007; Nashun et al, 2015), all of which are important considerations to fully mimic neurological disease states. Strategies are being developed to address each of these shortcomings, such as engrafting absent cell types including microglia, spatial patterning of signals and/or cells to control tissue architecture, bioengineering to introduce infiltrating structures for nutrient delivery, induced aging via targeted protein expression, and avoiding epigenetic erasure by bypassing the hiPSC stage with direct cell reprogramming (Vierbuchen et al, 2010; Miller et al, 2013; Quadrato et al, 2016; Di Lullo & Kriegstein, 2017; Soliman et al, 2017; Lovett et al, 2020; Garreta et al, 2021; Hofer & Lutolf, 2021). Finally, systematic studies implementing these strategies in concert with characterization by emergent technologies, ranging from transcriptomics to high-resolution imaging, will be critical in understanding and subsequently reducing organoid batch-to-batch variability (Quadrato et al, 2016; Di Lullo & Kriegstein, 2017; Garreta et al, 2021; Hofer & Lutolf, 2021). Overall, the hiPSC approach has undergone noteworthy growth with actionable improvements in modeling human neurological disease over the past 15 yr, and the results from applying such models have been proven immediately useful in deepening our understanding of cellular mechanisms driving AD pathologies.

### Modeling *APOE* ε4 risk in Alzheimer’s disease using hiPSC-derived cells

hiPSC-based model systems provide a platform to scrutinize cell type–specific functions that contribute to sporadic AD pathologies in a genotype-dependent manner. The amyloid hypothesis puts forth a neuron-centric view of AD etiology, where neurons do play an essential role as the main producers of Aß and are highly vulnerable to damage (De Strooper & Karran, 2016). However, the combination of hiPSC-based models and more refined characterization methods, including transcriptomic profiling, has enabled the field to study and appreciate the profoundly interconnected roles of other brain cell types, together with neurons, in AD onset and progression (Lambert et al, 2013; De Strooper & Karran, 2016). These findings are summarized in Fig 1.

### Neurons

We begin by considering *APOE*-dependent outcomes in the context of hiPSC-derived neurons. In general, *APOE* ε4 neurons produce more Aß-42 and have higher p-tau levels in comparison to *APOE* ε3 neurons (Duan et al, 2014; Lin et al, 2018; Wang et al, 2018; Wadhwani et al, 2019; Lee et al, 2021). This finding on amyloid extends to hiPSC-derived *APOE* ε4 neurons, generating more Aß aggregates upon transplantation into human *APOE* ε4- (as compared with *APOE* ε3-) knockin mice models (Najm et al, 2020). The field has reached some consensus that *APOE* ε4 represents a gain of toxic function rather than a loss of function, where *APOE*-deficient neurons display similar Aß and p-tau pathological phenotypes to those expressing *APOE* ε3 (Shi et al, 2017; Wang et al, 2018). Notably, the heightened Aß production was only observed in *APOE* ε4 human, not mouse, neurons, highlighting the species difference in *APOE* isoform–dependent Aß metabolism (Wang et al, 2018). Although Wang et al (2018) established that a small-molecule ApoE4-structure corrector could resolve these AD-related neuronal phenotypes, treatment at the ApoE protein level has yet to be realized in the clinical space (Wang et al, 2018). Transcriptomic analysis of isogenic *APOE* ε3 versus ε4 neurons (derived from a non–AD-affected individual and gene-edited ε3 to ε4) has revealed broad changes in expression of genes involving synaptic function in neurons (Lin et al, 2018). Specifically, *APOE* ε4 neurons in culture exhibit early maturation, elevated synaptic activity, and an increase in both the number of synapses and early endosomes, with a corresponding increase in secretion of the more aggregation-prone Aß-42 peptide (Lin et al, 2018; Meyer et al, 2019). Conversion of *APOE* ε4 to ε3 in hiPSCs from a sporadic AD patient attenuated many of these AD-related phenotypes in the differentiated neurons (Lin et al, 2018). In contrast, transcriptomic analysis of hiPSC-derived mixed cortical cultures in a different study revealed a lack of *APOE*-dependent differentially expressed genes related to neuronal maturation (TCW et al, 2022). Studies have also uncovered diverging effects dependent on the neuron subtype, demonstrating hyperexcitable glutamatergic neurons versus degeneration of GABAergic interneurons in culture (Lin et al, 2018; Wang et al, 2018). *APOE* ε4 in hiPSC-derived neurons has additionally been demonstrated to cause defective degradation pathways of autophagy and mitophagy (Fang et al, 2019). Taken together, *APOE* ε4 neurons suffer from increased Aß secretion and modulated Aß processing pathways, elevated p-tau levels, altered maturation resulting in augmented synaptic activity and increased electrical excitability, and endosomal and mitochondrial dysfunctions.

### Glia

Glial cells, which encompass astrocytes, microglia, and oligodendrocytes, provide critical metabolic, immune, and physical support to the brain. Framed by the amyloid hypothesis, *APOE* ε4–expressing hiPSC-derived glia have been repeatedly shown to develop trafficking defects that perturb cerebral Aß peptide oligomerization and effective clearance (Fernandez et al, 2019; Knopman et al, 2021; de Leeuw et al, 2022). From a combination of transcriptomics and cell culture experiments, *APOE* ε4 astrocytes demonstrate impaired Aß uptake compared with isogenic *APOE* ε3 astrocytes, aligning to the expected result of net higher extracellular Aß-42 concentration (Lin et al, 2018). Alternatively, astrocytic trafficking defects arising from *APOE* ε4 can also disrupt endocytosis in an Aß-independent manner. Further study of trafficking in hiPSC-derived astrocytes has established a compensatory functional connection between *APOE* ε4 and another AD risk factor, *PICALM*: although *APOE* ε4 expression was shown to cause defects in early endosomes that disrupted endocytic trafficking in astrocytes, increasing expression of *PICALM* was able to rescue the system (Narayan et al, 2020). Mechanistically, *APOE* ε4 astrocytes produce significantly less ApoE protein than ε3 (Lin et al, 2018), and this protein remains in a hypolipidated state (Zhao et al, 2017), in agreement with previous studies in human tissue and mouse models (Mooijaart et al, 2006; Shi et al, 2017). These ApoE4-containing lipoprotein particles, in turn, possess diminished binding efficiency to clear Aß (Kim et al, 2009). Some evidence suggests that ApoE isoform-dependent effects on amyloid clearance chiefly stem from competition for the same receptor-mediated removal pathways from the brain rather than direct interaction (Holtzman et al, 2012; Verghese et al, 2013).

Recent work addressing *APOE*-based AD risk has consistently demonstrated the dysregulation of key lipid pathways in hiPSC-derived *APOE* ε4 glia. As a primary function of ApoE is the transport of cholesterol, substantial effort has been dedicated to deciphering the cholesterol connection to AD. Lipid metabolism perturbed in *APOE* ε4 astrocytes at the transcriptomic level has been validated in culture, with *APOE* ε4 astrocytes exhibiting an accumulation of cholesterol both intracellularly and extracellularly in the media, suggesting dysregulated cholesterol metabolism (Lin et al, 2018). The work of TCW et al (2022) mainly corroborates these findings, where hiPSC-based *APOE* ε4 astrocytes and microglia feature elevated cholesterol synthesis and accumulation, similarly validated through transcriptomic profiling with corresponding in vitro experiments (TCW et al, 2022). The authors suggest a mechanism in which lysosomes sequester the elevated free cholesterol away from the endoplasmic reticulum, causing the cell to falsely sense low intracellular cholesterol concentration. In turn, this miscommunication induces the glial cell to up-regulate de novo cholesterol biosynthesis and decrease cholesterol efflux. Importantly, these effects are only seen in human, not mouse, glial cells, underscoring the utility of hiPSC-based models (TCW et al, 2022). This putative cholesterol sequestration mechanism is supported by another study that applied proteomic and lipidomic analyses to characterize *APOE* genotype-dependent changes in hiPSC-based astrocytes (de Leeuw et al, 2022). However, the reduced cholesterol efflux observed by de Leeuw et al (2022) and TCW et al (2022) renders the increased cholesterol level in the media measured by Lin et al (2018) counter-intuitive, pointing to the complex metabolic dysregulations occurring in *APOE* ε4 astrocytes that require further study. Overall, each of these conclusions highlights the disruption of net cholesterol flux. The apparent distinctions likely arise from experimental differences in parameters such as incubation timings, cell media compositions, and methods of quantification. Bulk media measurements grant a valuable view into cholesterol load that neighboring cells may experience but only a snapshot of net accumulation that is a sum of dynamic processes including biosynthesis, efflux, influx, and turnover. hiPSC donor-specific differences and the number of hiPSC lines under study introduce the added factor of genetic heterogeneity between individuals.

In addition to cholesterol, hiPSC-based *APOE* ε4 astrocytes demonstrate broad lipid imbalances, including accumulation of unsaturated triacylglycerides within intracellular lipid droplets (Sienski et al, 2021). Such imbalances cause the astrocytes to be more sensitive to nutritional conditions or exogenous lipid stress. Promoting phospholipid synthesis via choline supplementation of culture medium can avert such lipid droplet accumulation and restore lipid homeostasis. These findings support the manipulation of glial lipid metabolism through exogenous supplementation (i.e., dietary changes) as a therapeutic strategy to alleviate *APOE* ε4-associated disease risk.

Building from these findings ascertained from astrocytes alone, another *APOE* ε4-induced feature that can be modeled with hiPSC systems is the disrupted metabolic coupling between neurons and astrocytes. From previous animal work, toxic fatty acids produced during periods of neuronal hyperactivity are shunted to astrocytes via lipoprotein particles of which ApoE is a constituent (Liu et al, 2015, 2017; Fernandez et al, 2019; Ioannou et al, 2019). Astrocytes subsequently store these fatty acids in lipid droplets. However, *APOE* ε4 both reduces the transport efficiency from neurons to astrocytes and diminishes the proficiency of astrocytes degrading neuronal lipids, resulting in compromised neurotrophic support (Qi et al, 2021). Similarly, hiPSC-derived *APOE* ε4 astrocytes in co-culture with neurons are less effective in supporting neuronal survival and synaptogenesis, thus jeopardizing neuronal health (Zhao et al, 2017). Another study finds that *APOE* ε4 astrocytes oversupply cholesterol to neurons, resulting in more neuronal lipid rafts to which APP and its processing secretases localize, culminating in higher Aß generation from neurons (Lee et al, 2021) (in agreement with a study done in mouse cells [Wang et al, 2021]). Here, as noted regarding astrocyte monocultures above, perturbations in net cholesterol flux can negatively impact surrounding cells and may implicate combined effects of cholesterol efflux, influx, and turnover. Of note, the faulty lipid transport capabilities of ApoE4 are exacerbated in the aging brain, in comparison with the young *APOE* ε4 carrier brain that seemingly has compensatory mechanisms to cope with deficient neurotrophic support from astrocytes (Fernandez et al, 2019). Adapting hiPSC-based cultures to better capture these aging effects and ApoE4-mediated disruption of this neuron-supportive function will provide a clearer picture of the nature and consequences of ApoE4-mediated lipid dysregulation.

ApoE is primarily regarded as a lipid transport protein originating from astrocytes, yet the expanding transcriptomic analyses of human tissue samples have identified many AD-driven changes in gene expression within microglia (Mathys et al, 2019; Bellenguez et al, 2022). In particular, transcriptomic analysis of the prefrontal cortex from AD patients has revealed a concomitant up-regulation of *APOE* in microglia and down-regulation in astrocytes, emphasizing the cell type–specific effects of the gene (Mathys et al, 2019). The recent addition of protocols to derive microglia from hiPSCs has now enabled in vitro modeling of this transcriptomic data. Studying hiPSC-derived microglia is particularly advantageous because of their highly reactive nature and difficulty to transfect that limits the successful application of viral techniques (Maes et al, 2019; Victor et al, 2022). Advances in understanding microglial roles in AD will benefit from recent developments in chimeric models that entail grafting, for example, iPSC hematopoietic progenitors onto humanized, immune-deficient mice, resulting in differentiation into microglia that acquire appropriate human microglial gene signatures and responsive behaviors (Hasselmann et al, 2019). Such models acknowledge the profoundly sensitive nature of microglia to their local environment and are able to correct for the transcriptomic deficiencies that microglia develop in isolation in vitro (Hasselmann et al, 2019; Mancuso et al, 2019; Svoboda et al, 2019; Xu et al, 2020; Claes et al, 2021).

*APOE* ε4 hiPSC-based microglia are reported to exhibit inflammatory gene activation and associated phenotypes, adopting distinct morphologies with impaired phagocytosis of extracellular Aß aggregates (Lin et al, 2018). Another study on hiPSC-derived microglia determined that the *APOE* ε4 genotype compromised phagocytosis, reduced migration, increased proinflammatory cytokine secretion, and led to defective glycolytic and mitochondrial metabolism (Konttinen et al, 2019). Collectively, glial activation often arises as a consequence of the *APOE* ε4 genotype, revealed by analysis of transcriptomic changes together with measurement of secreted proinflammatory chemokines and cytokines (Lin et al, 2018; de Leeuw et al, 2022; TCW et al, 2022). Inflammation is often considered a nonspecific hallmark of neurodegeneration (Krasemann et al, 2017; Butovsky & Weiner, 2018) and in the case of AD, is seemingly connected with and induced by other pathways, such as lipid dyshomeostasis. Indeed, the metabolic shift associated with activation and inflammation includes the accumulation of neutral lipids and lipid droplets, reminiscent of the baseline lipid state in *APOE* ε4 cells (Sienski et al, 2021; Victor et al, 2022).

Although some work has been done with microglia in co-culture systems, there remains a need for more hiPSC-based studies that elucidate the crosstalk between microglia and other brain cell types. Recently, Victor et al (2022) investigated the cellular interactions between hiPSC-derived neurons and microglia as a function of the *APOE* genotype (Victor et al, 2022). Interestingly, soluble signaling from neurons provoked *APOE* ε4 microglia to enter a unique metabolic program, leading to the accumulation of neutral lipid droplets because of impaired lipid catabolism, in conjunction with decreased uptake of extracellular fatty acids because of the already saturated intracellular lipid machinery. In turn, this response shifted microglia away from their prototypical immune surveillance functions and weakened the neuron-microglia coupling required for microglia to adequately respond to modulations in neuronal activity, to the extent of microglia even disrupting coordinated neuronal activity. This cascade ultimately resulted in an intensified pro-inflammatory response, in line with *APOE* ε4 expression in microglia generally being associated with inflammation (Lin et al, 2018; Fernandez et al, 2019; Yamazaki et al, 2019). In this study, neurons were found to express *APOE* ε4, as expected in hiPSC-derived cells that are often in a stressed state. Toward therapeutic intervention, pharmacological blocking of lipid synthesis in *APOE* ε4 microglia was able to remediate these intracellular lipid droplets and restore microglial homeostasis.

Finally, the roles of oligodendrocyte dysfunction and myelin degeneration in AD pathology are becoming increasingly appreciated (Akay et al, 2021; Blanchard et al, 2022). Transcriptomic analysis of prefrontal cortex tissue has identified oligodendrocytes as one of the most altered cell types in AD (Mathys et al, 2019; Lau et al, 2020). The advent of protocols to derive oligodendrocyte precursor cells from hiPSCs offers an avenue to hiPSC-based models of this cell type (Douvaras et al, 2014; Penney et al, 2020; Akay et al, 2021). Model systems have been developed to exemplify in vitro myelination with neuronal co-cultures or artificial axons, as reviewed elsewhere (Blanchard et al, 2022), providing promising future directions to the study of myelin in the context of AD-relevant risk factors such as *APOE* ε4.

### Three-dimensional co-culture systems

Some findings as outlined above have been built upon to capture sporadic AD in organoid culture. This is exemplified by Lin et al (2018), in which the study was extended to model the *APOE* ε4–dependent defects in organoids containing neurons and astrocytes. In line with the neuron monoculture results (after 6 wk), *APOE* ε4 organoids exhibited more extracellular Aß accumulation and elevated tau phosphorylation (after 6 mo in culture; in comparison, the corresponding fAD organoid model requires the shorter time course of 2–3 mo to display a similar phenotype [Raja et al, 2016]). Crucially, this demonstrates that *APOE* ε4 alone is sufficient to cause AD hallmarks in cerebral organoids. Also using organoids, these findings have subsequently been validated and provided with a molecular mechanism implicating impaired function of the transcriptional regulator REST (Meyer et al, 2019). REST serves as a key repressor of neuronal differentiation that is normally induced by aging yet was found via gene network analysis to exhibit a loss of function in both sporadic AD and *APOE* ε4 neural cells (Meyer et al, 2019). Reduced REST function arises from its decreased nuclear localization and altered chromatin binding, with associated nuclear lamina disruption (Meyer et al, 2019). In turn, neuronal maturation processes are up-regulated, resulting in the previously described phenotype of premature neuronal differentiation, reduced progenitor cell renewal, accelerated synapse formation, and heightened electrical excitability (Meyer et al, 2019). Of note, accelerated differentiation was not reversed by inhibiting Aß generation and appeared before increased levels of tau phosphorylation; therefore, REST dysfunction may precede the canonical amyloid and tau pathologies (Meyer et al, 2019). Taken together, reduced REST function leading to a depleted progenitor pool and disrupted neural circuit formation may contribute to AD onset. A related study using AD patient hiPSC-derived cerebral organoids discovered that although *APOE* ε4 likely leads to early neuronal maturation, it also exacerbates synaptic loss in mature cerebral organoids (week 12) (Zhao et al, 2020). As such, enhanced differentiation and maturation of neurons in the early stages of development is posited to induce a corresponding mechanistic exhaustion and depleted cognitive reserve that accelerates neurodegeneration in the late disease stages.

### Blood–brain barrier cells

The blood–brain barrier (BBB) is comprised of endothelial cells, pericytes, and astrocytes that play an indispensable role in nutrient and oxygen delivery to and waste removal from the brain. BBB dysfunction and breakdown is observed across many neurodegenerative diseases, including AD, with a dependency on *APOE* isoform (Bell et al, 2012; Montagne et al, 2020). Blanchard et al (2020) have newly developed a three-dimensional, in vitro BBB (iBBB) composed of hiPSC-derived endothelial cells, pericytes, and astrocytes to model the effect of *APOE* ε4 on cerebral amyloid angiopathy, a condition in AD where amyloid deposits along the brain vasculature (Blanchard et al, 2020). Upon exposure to conditioned media from familial AD neuronal culture as the source of Aß, *APOE* ε4 iBBB cultures exhibited significantly higher amyloid accumulation along the blood vessels compared with risk-neutral *APOE* ε3 cultures. Based on a combinatorial cell-type screen with complementary transcriptomic analysis, it was determined that up-regulated *APOE* ε4 expression by pericytes was the critical component necessary for the amyloid angiopathy phenotype to occur. Further analysis revealed that dysregulation of nuclear factor of activated T cells (NFAT)–calcineurin signaling mediated the up-regulation of ApoE4 in pericytes, therefore increasing amyloid deposition and BBB disruption and providing a potential therapeutic target. Looking forward, microfluidic-based co-culture platforms are excitingly moving toward fully hiPSC-derived cell models of the BBB with functioning blood vessels, offering a promising route to probe AD pathology as a function of the *APOE* ε4 genotype (Campisi et al, 2018; Shin et al, 2019; Hajal et al, 2022). More broadly, developing a fully hiPSC-derived functional brain tissue with an integrated BBB is of high interest for studying the interplay between these vascular cells and other brain cell types.

### Outstanding challenges and future directions

Alzheimer’s disease continues to be a global health problem with severe psychological, social, and economic implications. A disease-altering treatment has yet to be realized. The repeated failures of hundreds of clinical trials over several decades to demonstrate efficacy in human AD patients has spurred the movement to develop more predictive disease models (Gonzalez et al, 2018; Penney et al, 2020). Although no single model has yet to holistically capture the complex AD etiology, advances in this platform-development space have proven useful (Lovett et al, 2020; Penney et al, 2020; Blanchard et al, 2022; Bubnys & Tsai, 2022). Animal models have enabled key contributions to understanding AD, though evolutionary differences render sole reliance on these systems difficult. The capacity to reprogram human fibroblasts into stem cells has begun to revolutionize the study of human disease. Recently developed hiPSC-based brain models provide an avenue to study the mechanisms underlying AD pathologies and drug responses in treating such pathologies in a genetically human background. In contrast to most other AD models, hiPSC systems do not necessitate exogenous overexpression of proteins to induce disease pathologies. Mounting evidence supports the use of hiPSC technology, together with human postmortem tissues and animal models, to build consensus within the field on the connection between genetic susceptibilities and consequent molecular mechanisms and cellular contributions.

Most of the hiPSC work modeling sporadic AD to-date has involved single- or few-cell-type cultures to derive fundamental understanding of the *APOE* risk factor function. Technologies merging multiple cell types and physiological features present in the actual human brain will be key in building next-generation in vitro platforms to study neurodegenerative disease. Moreover, this will enable the expansion from probing simplified genetic factors to more complex polygenic and/or environmental factors in driving AD risk (Cairns et al, 2020). Broadly, the study of *APOE* and other AD risk factors requires robustly validated hiPSC-derived brain models that accurately and reproducibly express relevant pathologies, potentially requiring multiple complementary models tailored to best address different biological questions (Blanchard et al, 2022). However, as we move closer to recapitulating the necessary biological complexity of the human brain, it is increasingly important to incorporate the ethical considerations of these models into the research itself (Farahany et al, 2018; Sawai et al, 2019; Garreta et al, 2021). Such models can serve as testing platforms toward the ultimate goal of rational interventions to treat or ideally prevent AD. Some work has established the translatability of highly uniform and homogeneous cerebral organoid models into high-throughput array screening platforms, with applications in discovering novel drug targets and testing candidate drugs to assess effective therapeutic intervention (Gonzalez et al, 2018; Park et al, 2021).

In the future, such hiPSC-based brain cell cultures will benefit from several technological developments, including integration of sensors to monitor dynamic molecular changes (Acarón Ledesma et al, 2019), engineering the extracellular milieu to support longer term culture of various cell types (Bretherton & DeForest, 2021; Hofer & Lutolf, 2021), and implementing spatiotemporal control to tune cell culture conditions in real time (Karimi et al, 2016; Lovett et al, 2020). Brain model platforms will be further enhanced by biological advances in incorporating an in vivo analogous immune component and perfusable vasculature (Park et al, 2018; Hajal et al, 2021; Blanchard et al, 2022). We anticipate hiPSC-based brain co-culture models to open avenues for intervention by revolutionizing AD drug development and testing toward a future cure.
