# Correction: Building in vitro models of the brain to understand the role of *APOE* in Alzheimer’s disease

**DOI:** 10.26508/lsa.202201845

**Published:** 2022-12-01

**Authors:** Rebecca L Pinals, Li-Huei Tsai

**Affiliations:** 1 Picower Institute for Learning and Memory, Massachusetts Institute of Technology, Cambridge, MA, USA; 2 Department of Brain and Cognitive Sciences, Massachusetts Institute of Technology, Cambridge, MA, USA; 3 Broad Institute of Harvard and MIT, Cambridge, MA, USA

## Abstract

Human induced pluripotent stem cell (hiPSC)–based models of the brain will be key to unraveling the role of APOE ɛ4 in the interconnected cellular changes underlying Alzheimer’s disease.

Article: Pinals RL, Tsai L-H (2022 Sep 27) Building in vitro models of the brain to understand the role of *APOE* in Alzheimer’s disease. Life Sci Alliance 5(11): e202201542. doi: 10.26508/lsa.202201542. PMID: 36167428.

In the initially published version of this article, a section in the Introduction reads:

In the amyloidogenic pathway, β-secretase first cleaves APP at the ectodomain, followed by γ-secretase at the intramembrane site, liberating the longer Aß-42 species. This contrasts with the physiologically normal pathway in which α- then γ-secretases consecutively cleave APP, shedding the shorter Aß-40 species.

This section should instead read:

In the amyloidogenic pathway, β-secretase first cleaves APP at the ectodomain, followed by γ-secretase at the intramembrane site, liberating Aß peptides including Aß-40 and Aß-42 (among other peptide lengths).

## Supplementary Material

Reviewer comments

