# Supplementary material for: Correction: Building in vitro models of the brain to understand the role of APOE in Alzheimer’s disease
Source: Life Sci Alliance. 2022 Dec 1;6(2):e202201845. doi: 10.26508/lsa.202201845 (PMC9717695; doi:10.26508/lsa.202201845)
Supplement: Reviewer comments [file LSA-2022-01845_review_history.pdf]

Correction: Building in vitro models of the brain to understand the role of APOE in Alzheimer's...

Li-Huei Tsai and Rebecca Pinals
DOI: https://doi.org/10.26508/lsa.202201845

Corresponding author(s): Li-Huei Tsai, Massachusetts Institute of Technology

Review Timeline:

	Submission Date:	2022-11-22
	Accepted:	2022-11-23

Scientific Editor: Eric Sawey, PhD

Transaction Report:

(Note: With the exception of the correction of typographical or spelling errors that could be a source of ambiguity, letters and reports are not edited. The original formatting of letters and referee reports may not be reflected in this compilation.)

November 22, 2022

RE: Life Science Alliance Manuscript #LSA-2022-01845

Dr. Li-Huei Tsai
Massachusetts Institute of Technology
Brain and Cognitive Sciences
43 Vassar Street
Cambridge, MA 02139

Dear Dr. Tsai,

Thank you for submitting your Correction entitled "Correction: Building in vitro models of the brain to understand the role of APOE in Alzheimer's Disease".

You can contact the journal office with any questions, contact@life-science-alliance.org

Sincerely,

Eric Sawey, PhD
Executive Editor
Life Science Alliance
http://www.lsajournal.org

November 22, 2022

RE: Life Science Alliance Manuscript #LSA-2022-01845

Dr. Li-Huei Tsai
Massachusetts Institute of Technology
Brain and Cognitive Sciences
43 Vassar Street
Cambridge, MA 02139

Dear Dr. Tsai,

Thank you for submitting your Correction entitled "Correction: Building in vitro models of the brain to understand the role of APOE in Alzheimer's Disease".

You can contact the journal office with any questions, contact@life-science-alliance.org

Sincerely,

Eric Sawey, PhD
Executive Editor
Life Science Alliance
http://www.lsajournal.org
